# RSPO4-CRISPR alleviates liver injury and restores gut microbiota in a rat model of liver fibrosis

**DOI:** 10.1038/s42003-021-01747-5

**Published:** 2021-02-18

**Authors:** Linghua Yu, Linlin Wang, Xiaojun Wu, Huixing Yi

**Affiliations:** 1 Center for Gastroenterology and Hepatology, Institute of Liver Diseases, The Affiliated Hospital of Jiaxing College, Jiaxing, Zhejiang Province PR China; 2 Department of Basic Medicine Sciences, School of Medicine, Zhejiang University, Hangzhou, Zhejiang Province PR China; 3 Intensive Care Unit, The Second Affiliated Hospital of Zhejiang University, Hangzhou, Zhejiang Province PR China

**Keywords:** Diseases, Gastroenterology

## Abstract

Wnt signaling dysfunction and gut dysbiosis may lead to liver fibrosis, yet the underlying mechanisms are not well elucidated. This study demonstrated the role of RSPO4, a Wnt signaling agonist, in liver fibrogenesis and its impact on the gut microbiome. RSPO4 gene in CCl_4_-induced fibrotic-liver rats was knockout by Clustered Regularly Interspaced Short Palindromic Repeats (CRISPR) system, with healthy rats served as the control. Tissue samples and hepatic stellate cells (HSCs) isolated from rats were examined for curative effect of RSPO4-CRISPR treatment. Fecal sample were collected and analyzed with 16 S rRNA sequencing. We found RSPO4-CRISPR relieved liver fibrosis in rats and reversed HSC activation. Further, results showed RSPO4-CRISPR tended to restore the microflora composition. Significance species between groups were identified. Bacteroides and Escherichia-Shigella were the key microbes in the model and negative group, whereas Lactobacillus, Romboutsia, and Lachnospiraceae NK4A136 group were abundant in the control. Notably, Bacteroidales S24-7 group and Ruminococcaceae UCG-005 were the significantly enriched in CRISPR group. We show that the microbiome of rats treated with RSPO4-CRISPR presents a trend towards the restoration of the original condition. Our findings pave a new way to evaluate the curative effect of liver fibrosis treatment.

## Abstract (web summary)

Yu et al. demonstrates the role of RSPO4, a Wnt signaling agonist, in liver fibrogenesis and its impact on the gut microbiome. By knocking out *RSPO4* gene in fibrotic-liver rat model, they show that it relieved liver fibrosis in rats and restored the microflora composition, highlighting its potential in liver fibrosis treatment.

## Introduction

Liver fibrosis, a reversible wound-healing response to various forms of liver injury, is an increasing worldwide health burden^1–3^. The advanced stage of liver fibrosis, known as cirrhosis, is a highly fatal disease with limited therapeutic options^4–6^. Thus, finding effective therapies and non-invasive detection of liver fibrosis in early stages is a tremendous medical challenge.

Gut microbiota has a direct impact on metabolism, immunity, and response to infection in humans. Liver disease is associated with gut dysbiosis and prior studies have revealed alterations in gut microbiota in patients with liver fibrosis and found that dysbiotic changes in the gut microflora contribute to the pathogenesis of liver disorder^7,8^. The liver receives nutrients from the intestinal circulation via the portal vein, making it the first target of the gut microbiota^9^. In healthy individuals, the intestinal mucosa acts as the “gut barrier” against the translocation of bacteria and microbial-derived products. Upon gut dysbiosis, the barrier dysfunction and alterations of the gut microbiota will cause liver injury and contribute to the progression of hepatic disease^10^.

Following liver injury, multiple intracellular signaling pathways drive hepatic stellate cell (HSC) activation, a crucial step in liver fibrogenesis. Numerous studies have shown that Wnt pathway activation is the central event of liver fibrogenesis^11^. Roof plate-specific spondin 4 (RSPO4), which is involved in a broad range of developmental and physiological processes, is a Wnt pathway potentiator^8,9^, and prior studies have shown that enrichment of RSPO4 induces epithelial proliferation and leads to enlargement of the mouse gastrointestinal tract^10,11^. Although we have previously demonstrated that RSPO1/2/3 is correlated with liver fibrosis^12^, the role of RSPO4 in liver fibrogenesis and its impact on the gut microbiome has not been well defined.

In the present study, we knocked out the RSPO4 gene in rats with CCl_4_-induced liver fibrosis using the Clustered Regularly Interspaced Short Palindromic Repeats (CRISPR) system. The impact of RSPO4 knockout on the gut microbiome was investigated with 16 S rRNA sequencing. This study aimed to identify a panel of significant gut microbial species associated with liver fibrosis and its regression to normal status by controlling the underlying causes, which could be used to evaluate the therapeutic effectiveness of liver fibrosis treatments.

## Results

### RSPO4-CRISPR relieves liver fibrogenesis in rats

**Fig. 1:**
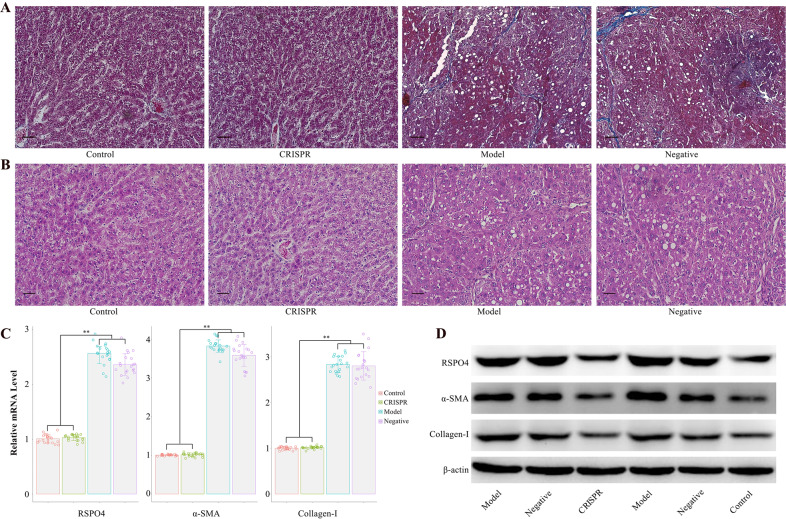
RSPO4-CRISPR relieve hepatic fibrosis in a rat model of CCl_4_-induced liver fibrosis. **A** Masson staining indicated the restoration of hepatic fibrosis in the CRISPR group (bar 50 μm, magnification ×200). **B** HE staining showed decreased vacuoles degeneration of hepatocytes in the CRISPR group (bar 50 μm, magnification ×200). **C** the mRNA level of RSPO4, α-SMA, and collagen-I in CRISPR group were significantly lower than the model group and were close to those of the control. **D** Western blot assay showed the protein expression of RSPO4, α-SMA, and Collagen-I in the CRISPR group were significantly lower than the model group and were close to those of the control. The CCl_4_-induced liver-fibrotic rats were noted as the model group, and the rats in the model group transfected with RSPO4-CRISPR were noted as the CRISRP group. Data represent the mean of three independent experiments, and error bars are the standard deviation of means. *p < 0.05 compared with the control, **p < 0.01 compared with the control.

To investigate the role of RSPO4 in liver fibrogenesis, a rat model of liver fibrosis induced by CCl_4_ was established (Fig. 1a, b). CCl_4_-induced liver-fibrotic rats (the model group) were selected for in vivo transduction of lentivirus-expressing RSPO4-CRISPR (the CRISPR group) and CRISPR-NC (the negative group). Normal rats without any treatment served as the control. Liver tissues of rats were analyzed by Masson staining and Hematoxylin and eosin (H&E) staining. Masson staining of the rat liver tissues revealed that RSPO4-CRISPR may slow down liver fibrogenesis (Fig. 1A). Notably, the staining results showed the liver tissues of the CRISPR group tended to return to a normal status (similar to the control), and there was no significant difference between the model group and the negative group. Similar pattern could also be found in the HE staining (Fig. 1B). The recovering of vacuoles degeneration in the liver tissues of the CRISPR group was observed, which indicated that the knockout of RSPO4 might relieve the liver tissue injury. Consistent with the above findings, the results of PCR test (Fig. 1C) showed the mRNA levels of RSPO4, fibrosis biomarkers (α-SMA and collagen-I) in the CRISPR group decreased significantly compared with the model group and the negative group (*p* < 0.01 for RSPO4, α-SMA, and collagen-I). A noteworthy finding was that the mRNA levels of RSPO4, α-SMA, and collagen-I in the CRISPR group are likely to close to those of the control, suggesting RSPO4-CRISPR may be able to reverse the progression of liver fibrosis. Protein expression of RSPO4, α-SMA, and collagen-I (Fig. 1D) was examined by western blot assay. Aligned with the results of RT-PCR, the protein expressions of RSPO4, α-SMA, and collagen-I were notably weakened in the CRISPR group compared with the model group and the negative group. Together, these findings suggest that RSPO4-CRISPR may relieve liver fibrogenesis in rats.

### RSPO4-CRISPR reverses HSC activation

Following hepatic injury, intracellular signaling will drive HSC activation, the central event of liver fibrogenesis. As RSPO4 is a potentiator of the Wnt pathway, we hypothesized that RSPO4 knockout would repress HSC activation. Activated HSCs isolated from the CCl_4_-induced liver-fibrotic rats (the model group) were transfected with a lentiviral vector expressing RSPO4-CRISPR (the CRISPR group) and CRISPR-NC (the negative group). Freshly isolated rat HSCs without any treatment served as a control.

**Fig. 2:**
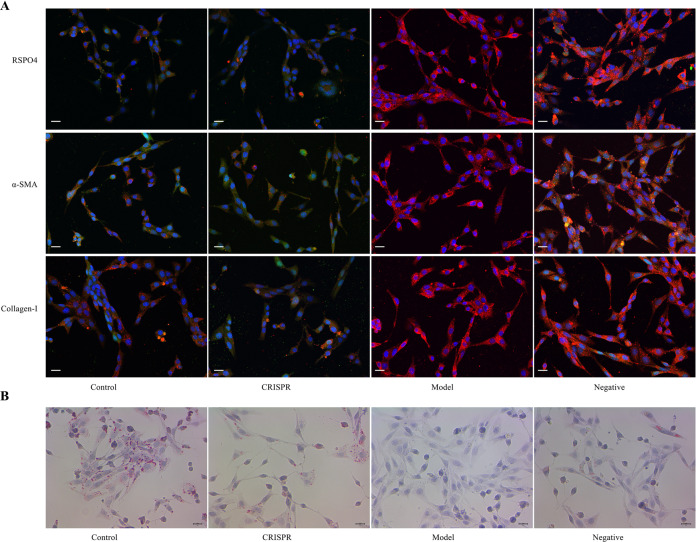
RSPO4-CRISPR suppresses HSC activation. **A** The results of immunofluorescence assay showed the protein expression of RSPO4, α-SMA, and collagen-I were lower in the CRISPR group than the model group and were close to those of the control (bar 20 μm, magnification ×400). **B**. Oil red O staining demonstrated the lipid droplets decreased in the CRISPR group compared with the model group (bar 20 μm, magnification ×400). HSCs isolated from the CCl_4_-induced fibrotic-liver rats were noted as the model group, cells transfected with RSPO4-CRISPR were noted as the CRISRP group and HSC isolated from normal rats served as the control. Data represent the mean of three independent experiments, and error bars are the standard deviation of means. *p < 0.05 compared with the control, **p < 0.01 compared with the control.

The single-guide RNAs (sgRNAs) inhibited the expression of RSPO4 via the introduction of a frameshift mutation. Protein expression of RSPO4 and fibrosis biomarkers α-SMA and collagen-I was examined by immunofluorescence assay (Fig. 2A). Results showed that the expression levels of RSPO4, α-SMA, and collagen-I in the CRISPR group were significantly decreased compared with the model and the negative control groups, and were very close to those of the quiescent HSCs (the control). Cytoplasmic lipid droplets were analyzed by oil red O staining. Consistently, results of the staining showed that lipid droplets in the CRISPR group were obviously restored compared with the model and showed nearly no difference from those of the quiescent HSCs (Fig. 2B). Taken together, these findings indicate the restoration to a quiescent status in the activated HSCs transfected with RSPO4-CRISPR.

**Fig. 3:**
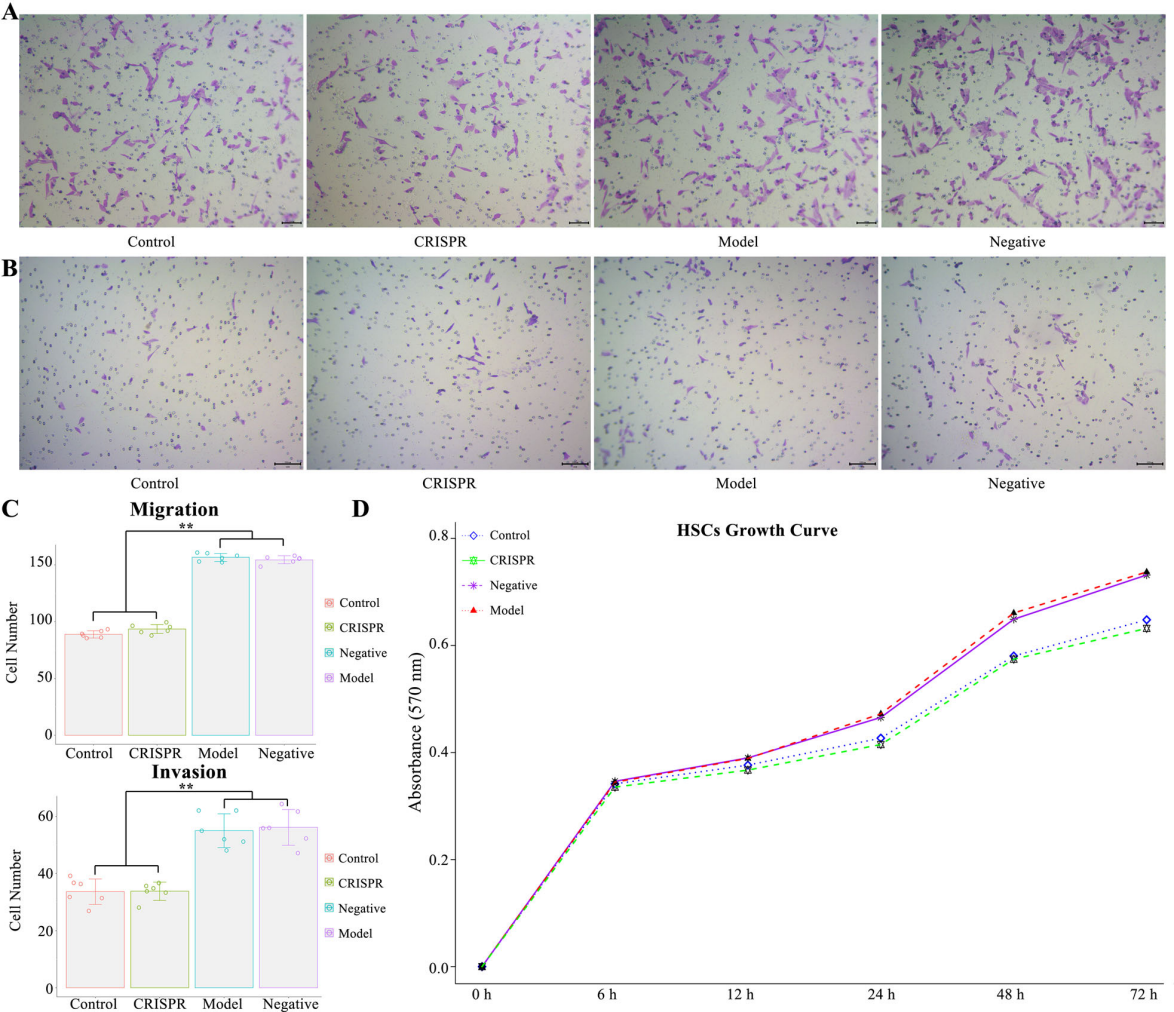
RSPO4-CRISPR represses HSC’s ability of migration, invasion, and growth. **A** Transwell assay showed RSPO4-CRISPR decrease HSCs’ ability of migration (bar 100 μm, magnification ×100). **B** Transwell assay showed RSPO4-CRISPR reduce HSCs’ ability of invasion (bar 100 μm, magnification ×100). **C** Cell number of both migration and invasion decreased significantly in the CRISPR group compared with the model group. **D** MTT assay showed RSPO4-CRISPR downregulated HSCs proliferation. HSCs isolated from the CCl_4_-induced fibrotic-liver rats were noted as the model group, cells transfected with RSPO4-CRISPR was noted as the CRISRP group and HSC isolated from normal rats served as the control. Data represent the mean of three independent experiments, and error bars are the standard deviation of means. *p < 0.05 compared with the control, **p < 0.01 compared with the control.

HSC activation causes extensive migration, invasion, and proliferation of the cells. HSC migration and invasion were tested by transwell assay. Results (Fig. 3A, C) showed the migration ability of the CRISPR group was significantly lower than that of the model and the negative group (*p* < 0.01), and there was no significant difference between the CRISPR group and the control. Consistently, results of the invasion assay (Fig. 3B, C) indicated that the invasion ability of the CRISPR group decreased significantly compared with that of the model and the negative group (*p* < 0.01), and there was no obvious difference between the CRISPR group and the control. Results of the MTT cell proliferation assay (MTT) showed that the growth rate of the CRISPR group was downregulated compared with that of the model and the negative group, and was close to that of the quiescent HSCs (Fig. 3D). In brief, these results suggest that RSPO4-CRISPR might reverse HSC activation.

### The community α-diversity of CRISPR group is significantly higher than that of the model and similar to control

Fecal samples of rats were collected from the CRISPR group (20 rats), the negative group (20 rats), the model (20 rats), and the control (20 rats). 16 S rRNA gene amplicon sequencing was performed on the DNA extracted from the 80 fecal samples. In all, 16 S rRNA sequences reads with high quality were obtained from the 80 samples with an average of 54,225 sequences per sample (the minimum of one sample was 48,977 reads and the maximum was 57,890 reads).

**Fig. 4:**
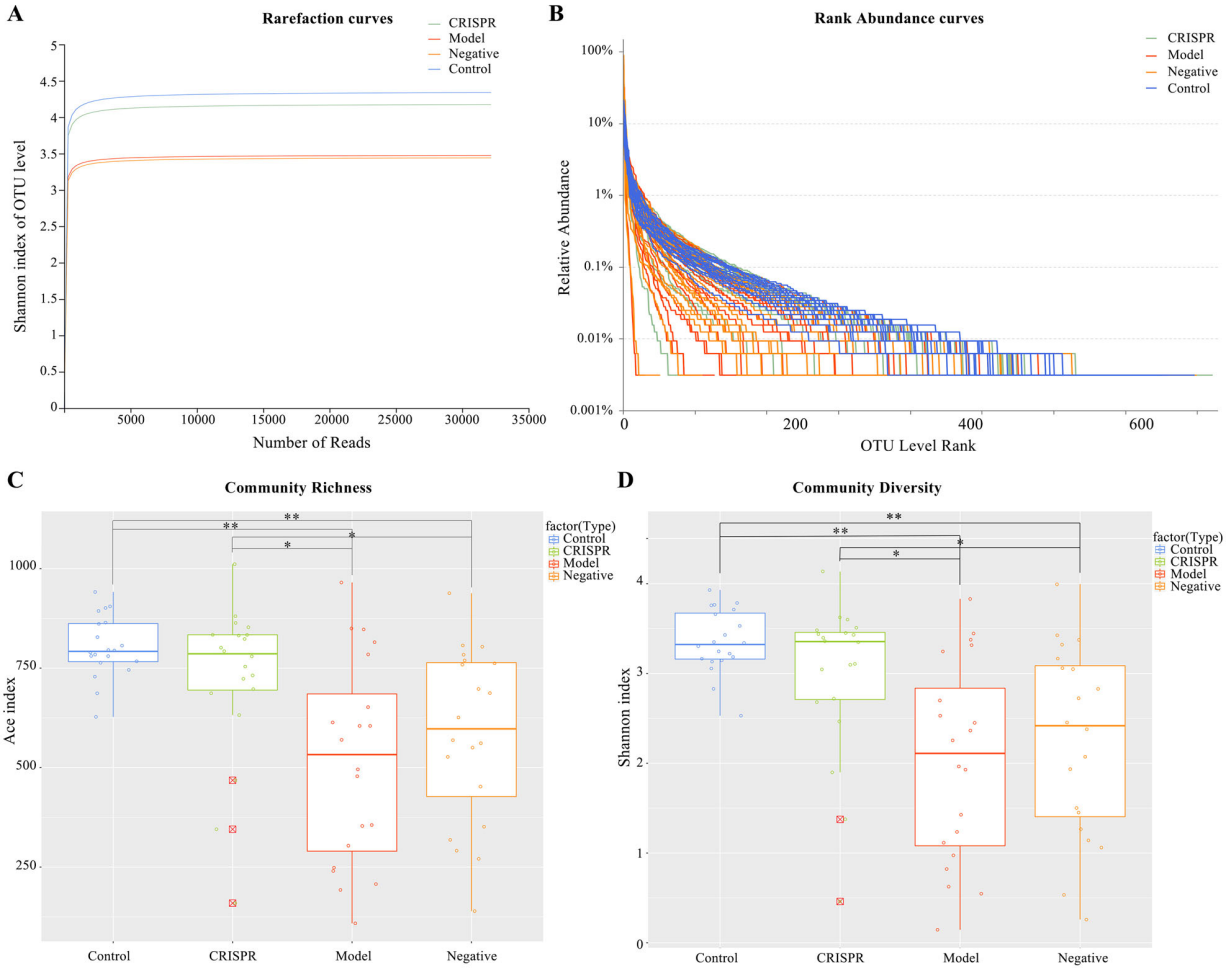
The community α-diversity of CRISPR group is significantly higher than that of the model group and similar to the control. **A** Rarefaction curves for Shannon index showed microbial diversity increases among the CRISPR group as compared WITH the model group and is similar to that of the control. **B** Rank abundance curves indicated the CRISPR group has higher richness and evenness of species than the model group and is close to that of the control. **C** The community richness of CRISPR group increased significantly compared to that of the model group and was close to that of the control. **D** The community diversity of CRISPR group was significantly higher than that of the model group and trended to approach that of the control. Fecal samples were collected from 32 rats with CCl_4_-induced liver fibrosis and 20 normal rat (the control). Fecal samples collected before injection of RSPO4-CRISPR were noted as the model group, and samples collected after the injection of RSPO4-CRISPR were noted as the CRISRP group.

Rarefaction curves for Shannon index tended to be smooth, which suggests a reasonable number of samples have been taken (Fig. 4A). Further, we observed from the rarefaction curves that the model group and the negative group had lower community diversity (Shannon index) than the CRISPR group and the control. Species richness and evenness of each sample was visualized with rank abundance curves (Fig. 4B). Aligned with the results of rarefaction curves, rank abundance curves indicated the CRISPR group showed higher species richness and evenness than the model group and was similar to those of the control. Alpha diversity (α-diversity) indexes of each sample were calculated. Results showed the community richness (Ace index) of the CRISPR group and the control were significantly higher than the model group (*p* < 0.01 for both the CRISPR group and the control) and the negative group (*p* < 0.05 for both the CRISPR group and the control) (Fig. 4C). Moreover, there was no statistical difference between the CRISPR group and the control in the Ace index (*p* = 0.12). Consistent with the community richness, results demonstrated the community diversity (Shannon index) of the CRISPR group and the control were significantly higher than the model group (*p* < 0.01 for both the CRISPR group and the control) and the negative group (*p* < 0.05 for both the CRISPR group and the control) (Fig. 4D). No statistical difference in Shannon index was found between the CRISPR group and the control (*p* = 0.48). Together, the above findings suggested the gut microbiota diversity of the CRISPR group increased compared with the model group and tended to be similar to the control.

### Liver fibrosis leads to gut microbiota dysbiosis, and RSPO4-CRISPR tends to restore the microflora composition to an equilibrium status, similar to the control

**Fig. 5:**
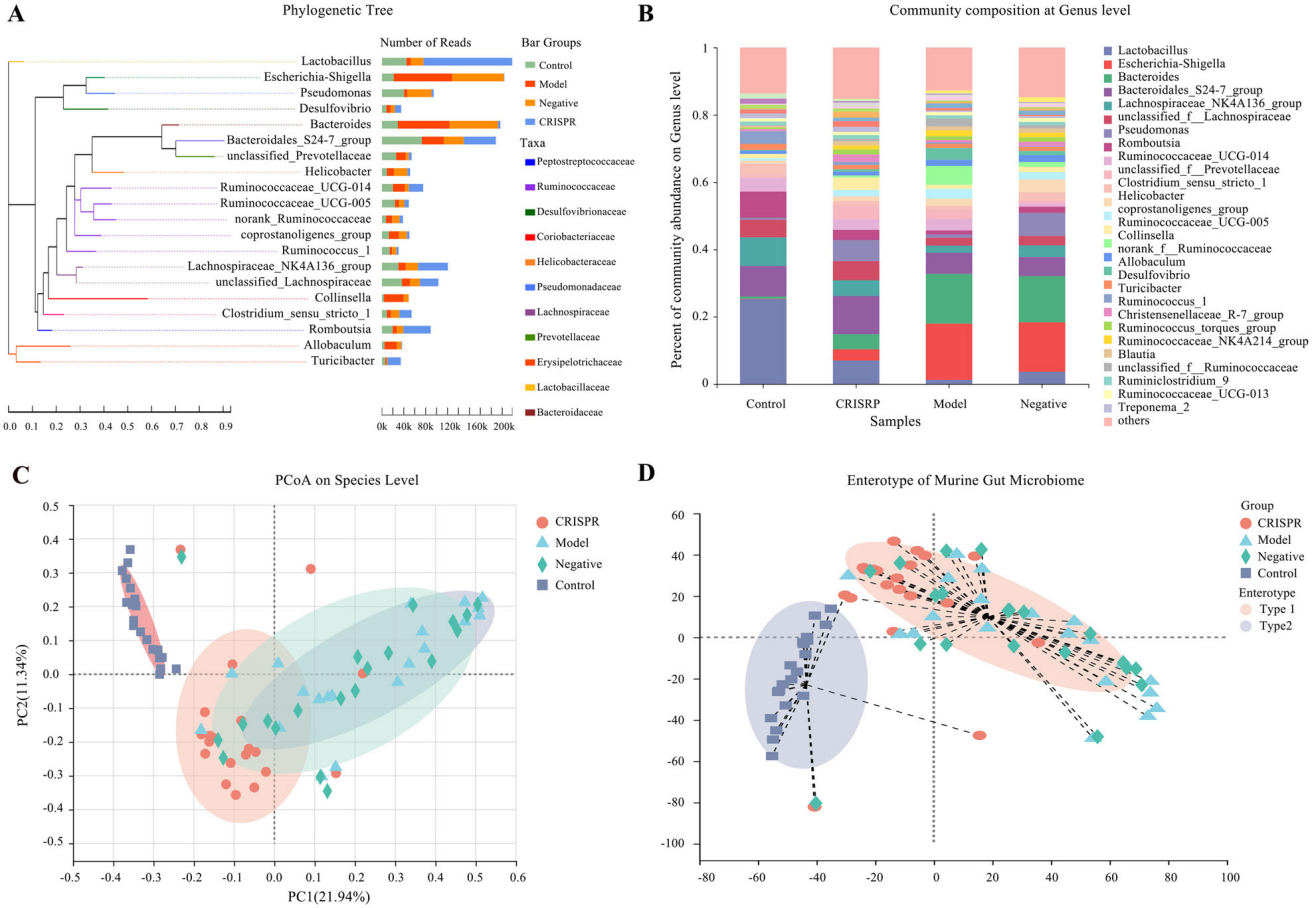
The gut microbiota composition of the CRISPR group tends to restore to an equilibrium status, similar to the control. **A** Phylogenetic tree of the samples with number of reads per genus showed *Escherichia*–*Shigella* has the highest abundance, followed by *Bacteroides*, Bacteroidales S24-7 group, and *Lactobacillus*. **B** Microbial community composition of the CRISPR group was apparently differed from that of the model group and was close to that of the control. **C** Samples were presented on a PCoA plot at species level and were grouped by eclipses with 95% confidence. Three distinguishable groups were identified, the CRISPR group was intersected with the model group and the control. **D** Two cognizable enterotypes were identified, type 1 mainly consisted of the model group and type 2 mostly included the control. Interestingly, samples in the CRISPR group belonged to both type 1 and type 2. Fecal samples were collected from 32 rats with CCl_4_-induced liver fibrosis and 20 normal rat (the control). Fecal samples collected before injection of RSPO4-CRISPR were noted as the model group, and samples collected after the injection of RSPO4-CRISPR were noted as the CRISRP group.

To reveal the phylogenetic relationship of taxonomy in the gut microflora of rats, a phylogenetic tree with number of reads per genus was constructed (Fig. 5A). Overall, results showed *Lactobacillus* (238,341 reads) had the highest abundance, followed by *Escherichia*–*Shigella* (223,949 reads), *Bacteroides* (216,570 reads), and Bacteroidales S24-7 group (208,292 reads).

According to the results of taxonomic analysis, the community composition of the model group, the negative group, the CRISPR group, and the control at the genus level was obtained (Fig. 5B). Results showed a distinct difference in gut microbiota community structure among groups. Overall, the model and the negative group shared a similar community composition pattern. *Escherichia*–*Shigella* had the highest abundance in the model group (16.7%) and the negative group (14.7%), and was followed by *Bacteroides* (14.8% in the model group and 13.7% in the negative group). On the other hand, *Lactobacillus* (25.2%), Bacteroidales S24-7 group (9.1%), Lachnospiraceae NK4A136 group (8.5%) were the dominant genera in the control. Similarly, Bacteroidales S24-7 group (11.3%), *Lactobacillus* (7%), and unclassified Lachnospiraceae (5.7%) were prevalent in the CRISPR group. Interestingly, the community composition plot showed that the CRISPR group had a different gut microbiota structure than the model group and tended to be similar to the control.

Multivariate analyses were applied to illustrate the overall community structure of gut microbiota among all fecal samples on the species level. Principal coordinates analysis (PCoA) was performed to visualize the difference in bacteria composition among groups (Fig. 5C). The first two components accounted for 33.28% (PC1: 21.94%, PC2(11.34%) of the total variation. Four distinct groups that represented the model group, the negative group, the CRISPR group, and the control were identified. The control was obviously distinct from the model group and the negative group, and the CRISPR group intersected with the model group and the control.

By clustering the samples with similar dominant microflora structures into one class, the microbiota typing analysis revealed two distinct enterotypes: type 1 and type 2 (Fig. 5D). Type 1 mainly consisted of samples from the model group, the negative group, and the CRISRP group, whereas type 2 mostly included samples from the control. Consistent with the findings in the PCoA analysis, samples from the CRISPR group were distributed in both type 1 and type 2. Together, the above findings showed the gut microbiota composition of the model group is apparently different from that of the control with lower diversity, which was consistent with previous studies reporting that liver fibrosis leads to gut microbiota dysbiosis^12^. Interestingly, stool samples from the CRISPR group showed that gut microbiota diversity increased compared with the model group, and the microflora composition tended to revert to an equilibrium status, similar to the control.

### Identification of species that significantly differ between the CRISPR, model, and control groups

**Fig. 6:**
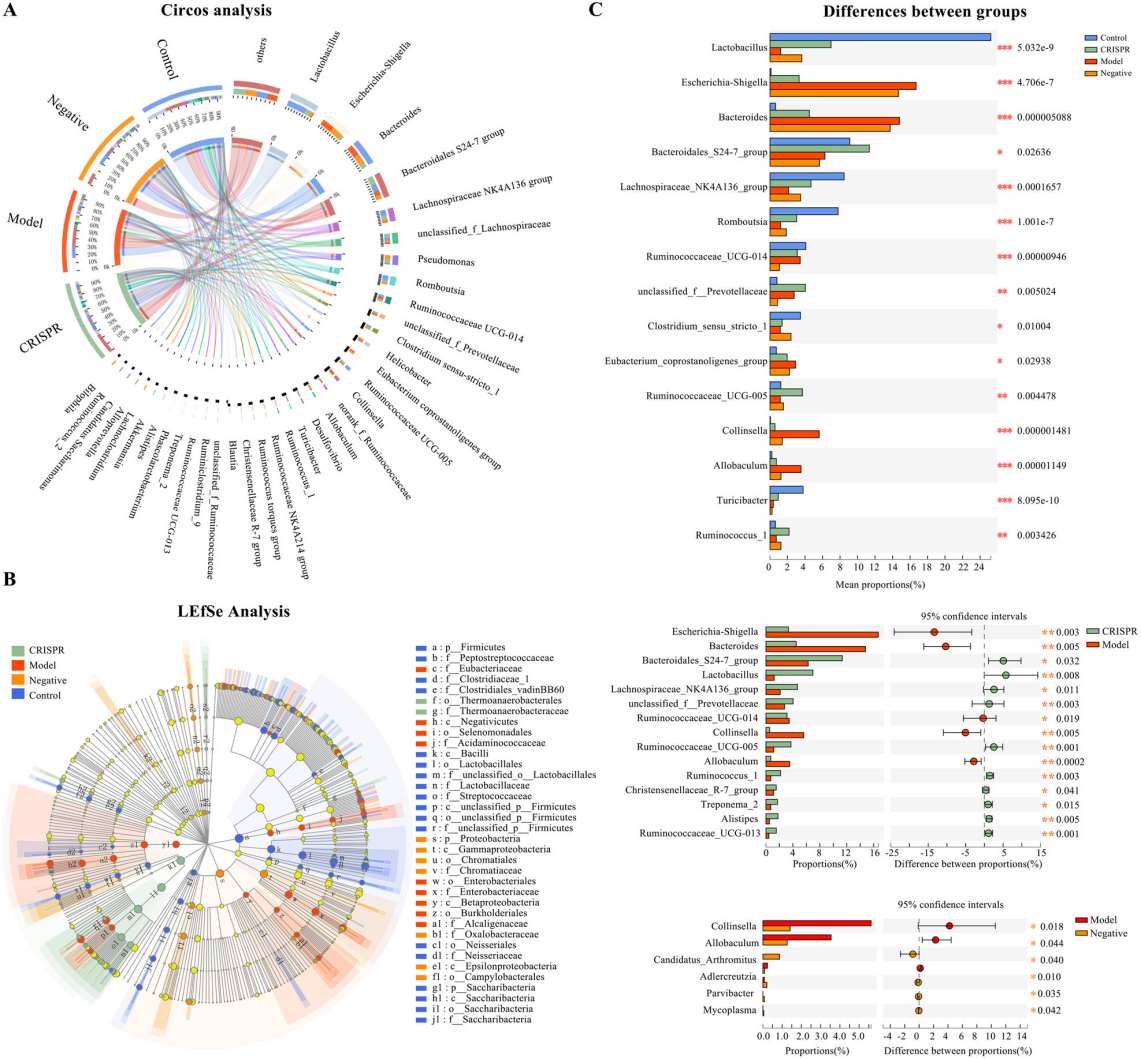
Species that significantly differ between the CRISPR group, the model group, and the control. **A** Circos analysis presented the distribution of the dominant species in the CRISPR group, the model group, and the control. **B** Kruskal–Wallis *H* test showed the abundance of several species were significantly different between the CRISPR group, the model group, and the control. *Lactobacillus* was significantly enriched in the control, Bacteroidales S24-7 group was high in the CRISPR group, and species *Escherichia*–*Shigella*, *Bacteroides*, and Collinsella were dominant in the model group. **C** The LEfSe results were aligned with those of the significant differences analysis between groups. Fecal samples were collected from 32 rats with CCl_4_-induced liver fibrosis and 20 normal rat (the control). Fecal samples collected before injection of RSPO4-CRISPR were noted as the model group, and samples collected after the injection of RSPO4-CRISPR were noted as the CRISRP group.

The corresponding relationships between the abundance of species and samples were analyzed by Circos plot (Fig. 6A). The co-occurrence graph clearly demonstrated the proportion of dominant species in each group (the model group, the negative group, the CRISPR group, and the control) and the distribution proportion of groups in the predominant species. *Lactobacillus*, Bacteroidales S24-7 group, *Escherichia*–*Shigella*, *Bacteroides*, Lachnospiraceae NK4A136 group, unclassified Lachnospiraceae, *Romboutsia*, and Ruminococcaceae UCG-014 were the predominant species in samples at the genus level. *Escherichia*–*Shigella* (the model group: 48%, the negative group: 42%, the CRISPR group: 9.6%, the control: 0.34%) and *Bacteroides* (the model group: 44%, the negative group: 41%, the CRISPR group: 13%, the control: 2%) were most abundant in the model and negative group, whereas *Lactobacillus* (the model group: 3.3%, the negative group: 9.7%, the CRISPR group: 19%, the control: 68%), Lachnospiraceae NK4A136 group (the model group: 11%, the negative group: 19%, the CRISPR group: 25%, the control: 45%) and *Romboutsia* (the model group: 9%, the negative group: 13%, the CRISPR group: 22%, the control: 56%) was predominant in the control. Bacteroidales S24-7 group (the model group: 19%, the negative group: 17%, the CRISPR group: 35%, the control: 28%) and unclassified Lachnospiraceae (the model group: 15%, the negative group: 17%, the CRISPR group: 35%, the control: 33%) were enriched in both the CRISPR group and the control.

The significant differences between the groups were analyzed via Kruskal–Wallis *H* test (Fig. 6B). Results were consistent with those of the Circos analysis. Several significant species were identified. *Escherichia*–*Shigella* and *Bacteroides* (*p* < 0.01) were significantly enriched in the model and negative group, Bacteroidales S24-7 group and Lachnospiraceae NK4A136 group were abundant in both the CRISPR group and the control (*p* < 0.01), and *Lactobacillus* and *Romboutsia* were predominant in the control (*p* < 0.01). Compared with the model group (Wilcoxon rank-sum test), genera *Escherichia*–*Shigella*, *Bacteroides*, and Collinsella (*p* < 0.01) were decreased in the CRISPR group, whereas Bacteroidales S24-7 group, *Lactobacillus*, and Lachnospiraceae NK4A136 group were increased in the CRISRP group. Notably, there was almost no difference between the model and the negative group.

To identify bacteria that were specific for the groups, linear discriminant analysis of the fecal microbiota was performed (Fig. 6C). LEfSe (Linear discriminant analysis Effect Size) results were aligned with those of significant differences analysis. Overall, the abundances of 94 genera were significantly different between the groups. Twenty-three genera were more abundant in the model group, including *Escherichia*–*Shigella* (LDA = 4.88), *Bacteroides* (LDA = 4.83), Collinsella (LDA = 4.45), Allobaculum (LDA = 4.18), and Blautia (4.06), which indicated that these bacteria positively responded to the liver injury. Besides, *Lactobacillus* (LDA = 5.06), *Romboutsia* (LDA = 4.51), Lachnospiraceae NK4A136 group (LDA = 4.49), and Ruminococcaceae UCG_014 (LDA = 4.17) were higher in the control. Interestingly, 21 genera were significantly enriched in the CRISPR group, including Ruminococcaceae UCG_005 (LDA = 4.1), *Ruminococcus*_1 (LDA = 3.91), and Ruminococcaceae UCG_013 (LDA = 3.76), which suggested these bacteria showed a positive response to the RSPO4-CRISPR injection.

## Discussion

The gut microbiome has emerged as a critical participator in the pathophysiology of many diseases^7,8,10^. The gut microbiota promotes the development of intestinal diseases, as in the inflammatory bowel disease and recurrent *Clostridioides difficile* infections, via local effects^13^. Further, researches reveal the correlation between the gut microbiota and diseases at distant sites such as the liver and brain, via the gut–liver axis and gut–brain axis, respectively^14^. Prior studies have reported that liver disorders associate with an altered microbiome^15–18^, but few of them focused on the alteration of the microbiome before and after liver fibrosis treatment. In our study, liver fibrogenesis in rats was relieved by knockout of the RSPO4 gene with the CRISPR system. The gut microbiome of rats before and after the treatment was analyzed by 16 S rRNA sequencing. Data showed that liver fibrosis is accompanied by gut dysbiosis, with low community α-diversity, and pathogenic bacteria dominate the bacterial community. Conversely, the treatment of RSPO4-CRISPR tended to restore the gut microflora to an equilibrium status similar to that of the healthy rat. This study also identified a group of significant taxa associated with liver fibrosis and its regression to normal status by treatment with RSPO4-CRISPR. We propose that this panel of significant taxa with other indices, such as the α-diversity and enterotype, can be a potential surveillance index for the therapeutic effectiveness of liver fibrosis treatment.

This study employed the CRISPR system to determine the function of RSPO4 in liver fibrogenesis. CRISPR is a highly specific, extremely efficient, and low-cost genome editing technology that is well-suited for various cell types and organisms^19^. The specificity of CRISPR relies on a 20-nt guide sequence within the sgRNA, which will direct Cas9 nuclease to a 20-bp DNA target via Watson–Crick base pairing^20^. By merely designing the oligos encoding the 20-nt guide sequence, Cas9 can be re-directed toward almost any target of interest. This study designed and screened sgRNAs for RSPO4 gene knockout (RSPO4-CRISPR). The specificity of binding to the target RSPO4 gene was verified by E7T1 enzyme digestion and sequencing^21–23^.

Liver fibrosis is an increasing worldwide health problem. Cirrhosis, the advanced stage of liver fibrosis, is among the most common causes of mortality worldwide and a significant risk factor of hepatocellular carcinoma^4–6^. Fortunately, increasing evidence shows that liver fibrosis, and even cirrhosis with decompensation, can regress with proper treatment^24–27^. Thus, finding effective therapies to prevent and reverse liver fibrosis is a tremendous medical challenge. Intracellular events and signals induce the activation of HSCs, the central driver of liver fibrogenesis, which makes them the potential targets for anti-fibrotic treatments. Our in vivo and in vitro studies showed that RSPO4-CRISPR effectively represses HSCs activation and relives liver fibrosis in rats. These findings suggest RSPO4 could be a compelling target for early detection and therapy of liver fibrosis.

Studies indicate that liver disorders are accompanied by dysbiotic changes in gut microbiota^10^. Thus, we hypothesized that the alteration of the gut microbiome would reflect the reversion of liver fibrosis as a consequence of controlling the underlying causes. Fecal samples from fibrotic-liver rats were analyzed by 16 S rRNA sequencing. Results showed the fibrotic-liver rats have lower species richness and evenness than the normal rats, which was consistent with previous studies reporting that liver disease leads to gut microbiota dysbiosis^28^. The community richness (Ace index) and diversity (Shannon index) of the fibrotic-liver rats increased significantly after receiving the RSPO4-CRISPR treatment (CRISPR group) were comparable to those of the model and negative group, and were nearly the same as the control. The taxonomic analysis presented a distinct difference in the gut microbiota community structure before and after the RSPO4-CRISPR medication. Further, PCoA analysis visualized the difference in bacteria composition between groups. The model group was obviously apart from the control, and the CRISPR group was intersected with the model group and the control. A similar pattern was also found in the enterotype analysis. Together, the above findings showed that the gut microbiome of the fibrotic-liver rats differs from that of the normal rats with lower diversity. In addition, the microbiome of rats medicated with RSPO4-CRISPR presented a trend towards the restoration of the normal condition with increasing community α-diversity. These findings confirmed our hypothesis that the gut microbiome could reflect the reversion of liver fibrosis as a result of treatment with RSPO4-CRISPR.

To derive a panel of significant species for monitoring liver fibrosis and its regression to normal status, significant difference tests and LEfSe analyses were performed. In this context, data showed that *Lactobacillus* was predominant in the control group. This result was consistent with the previous report that *Lactobacillus* inhibits bacterial b-glucuronidase activity and associates with hepato-protection^29^. Members of the genus *Lactobacillus* are the most abundant microbes in the mammalian gastrointestinal tract, and help to restore gut barrier integrity and maintain a healthy intestine^30^. Numerous studies have indicated a probiotic role for *Lactobacillus* in the prevention and resolution of infectious disease, which makes it an essential biomarker representing a healthy gut microbiome^31^.

This study found that *Bacteroides* and *Escherichia*–*Shigella* are significantly enriched in the fibrotic-liver rats. *Bacteroides* species are opportunistic human pathogens and can cause life-threatening infections including bacteremia^32^. Previous studies reported that *Bacteroides* species are associated with a high-fat diet, and its abundance is significantly increased in nonalcoholic steatohepatitis, which is consistent with our data^28^. Similarly, members of *Escherichia*–*Shigella* are also potential opportunistic pathogens that cause a spectrum of diseases, including diarrhea, and are a significant cause of mortality and morbidity worldwide^33^. Notably, our study also indicates that genera belonging to Ruminococcaceae and *Ruminococcus* are specific to rats treated with RSPO4-CRISPR. Reports link Ruminococcaceae to a healthy liver status^34,35^ and negatively associate it with injury-related factors^36^. Collectively, the indices of these significant species, such as the composition ratio and LDA index, may reflect the fibrotic status of the liver, and even its regression to the healthy status.

In conclusion, this study revealed that RSPO4 gene knockout with the CRISPR system relieves the liver fibrogenesis in rats, and restores dysbiotic gut microbiota related to liver fibrosis to an equilibrium status similar to healthy rats. Further, a panel of significant species was identified, which has the potential to serve as a surveillance index for the therapeutic effectiveness of liver fibrosis treatment. Finally, this group of significant species provides therapeutic targets for fecal microbiota transplantation treatment, which will require further studies.

## Methods

**Fig. 7:**
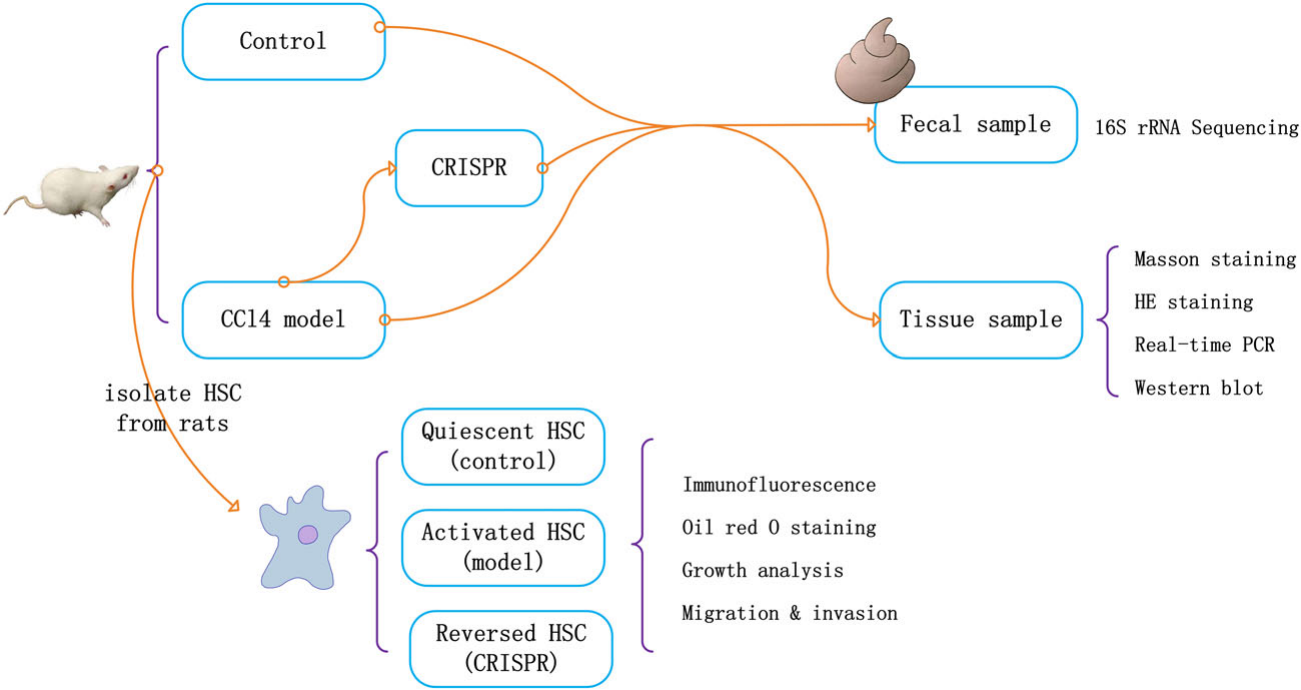
Study design. CCl_4_-induced fibrotic-liver rats (model group) were injected with lentivirus vectors expressing RSPO4-CRISPR (CRISPR group), with healthy rats served as the control. Fecal samples were collected and examined by 16 S rRNA sequencing. Tissue samples were analyzed by Masson staining, HE staining, PCR, and Western blot, respectively. Activated HSCs isolated from the CCl_4_-induced liver-fibrotic rats were transfected with lentivirus vector expressing RSPO4-CRISPR, with cells isolated from normal rats served as the control. HSC activation status was tested by immunofluorescence, Oil red O staining, Transwell assay, and MTT.

This study investigated the role of RSPO4 in liver fibrogenesis and its impact on the gut microbiome. An overview of the study design is shown in Fig. 7.

### Animals used

Male Sprague-Dawley (SD) rats (8–10 weeks, 250–300 g) were purchased from Shanghai SLAC Laboratory Animal Co Ltd (Shanghai, China). The animals were housed under standard animal laboratory conditions and supplied with laboratory chow and water. All individuals involved in animal research received instructions in experimental methods and the care, maintenance, and handling of animals. All institutional and national guidelines for the care and use of laboratory animals were followed. The protocol of the experiments was approved by the Committee on the Ethics of Animal Experiments of Jiaxing College (JUMC2018-014).

### The CCl_4_-induced fibrosis rat model

The CCl_4_-induced hepatic fibrosis model of the rat was induced by subcutaneous injection of 40% solution of CCl_4_ in olive oil at a dose of 5 ml/kg twice per week for four consecutive weeks. Eighty male SD rats (8–10 weeks old, 250–300 g of body weight) were randomly divided into two groups. The first group (*n* = 20) received no treatment and served as a normal control group. The rats with CCl_4_-induced fibrosis comprised the experimental group (*n* = 60). This group was then subdivided into those transfected with RSPO4-CRISPR (CRISPR group; *n* = 20); those transfected with a negative control lentivirus vector (CRISPR-NC group; *n* = 20); and those receiving no lentivirus (model group; *n* = 20). Rats were sacrificed by CO_2_ exposure and the entire livers were collected for HSC isolation, hematoxylin and eosin (H&E) staining, Masson staining, western blot analysis, and PCR.

### Histopathology

The left lobe of the rat livers was fixed in 4% paraformaldehyde for at least 24 h, embedded in paraffin, and cut into 5-µm-thick sections. Following hydration in a decreasing ethanol gradient (100%, 95%, 70 %, 50%, and pure water) for 5 min each, sections were deparaffinized and stained with H&E and Masson’s trichrome for 5 min at 37 °C. The stained sections were then observed under a light microscope (OLYMPUS BX43, Olympus Co., Japan).

### In vivo transduction of lentivirus

CCl_4_-induced liver-fibrotic rats were selected for injection of lentivirus vectors expressing RSPO4-CRISPR. In vivo transduction of lentiviruses (CRISPR-T3 and CRISPR-NC) was achieved through in situ injections of 30 μL of concentrated viral suspension with a viral titer of 1.0 × 10^8^ TU/mL lentiviral particles in phosphate-buffered saline (PBS); injections were conducted once. Three weeks after the dose, the animals were killed by CO_2_ exposure, and liver tissues were harvested.

### HSC isolation and culture

Hepatic stellate cells were isolated from the livers of normal male SD rats (250–300 g of body weight), as previously described^37^. In brief, rat liver was perfused in situ with Dulbecco’s Modified Eagle Medium (DMEM) (Gibco, United States) to purge the liver of blood. After digestion of the liver with pronase E (Sigma, United States) and collagenase IV (Sigma, United States), dispersed cell suspensions were layered by Nycodenz (Sigma, United States) density gradient centrifugation according to manufacturer’s protocol. The resulting upper layer contained the isolated HSCs. The purity of isolated HSCs was examined by phase-contrast microscopy, and viability based on trypan blue exclusion (purity >95%, viability >95%). The isolated HSCs were then cultured on uncoated plastic plates in DMEM (Gibco, United States) supplemented with 10% FBS (Gibco, United States) at 37 °C at a density of 1 × 10^5^ cells/cm^2^ for future research. HSCs were co-cultured with a lentiviral vector (CRISPR-T1 and CRISPR-NC, MOI = 50). 48 h after infection, cells were harvested for further study.

### sgRNA plasmid

Single-guide RNA (sgRNA) sequences targeting rat RSPO4 (Gene ID: 499918) were designed using online software (http://crispr.mit.edu). This study selected three sgRNA target sequences (target 1, 2, and 3) for RSPO4-CRISPR (Table 1, Supplementary Figure 1). CRISPR-NC served as the negative group.

**Table 1:** sgRNA sequences.

Name	Target	sgRNA
CRISPR-T1	GGCTTCATAAGTTCCAATCT	caccGGCTTCATAAGTTCCAATCT
		aaacAGATTGGAACTTATGAAGCC
CRISPR-T2	GAGCAGGAAGCAGTGTTGGT	caccGAGCAGGAAGCAGTGTTGGT
		aaacACCAACACTGCTTCCTGCTC
CRISPR-T3	GCTTTAACAGAGTACACTTA	caccGCTTTAACAGAGTACACTTA
		aaacTAAGTGTACTCTGTTAAAGC

### Immunofluorescence assays

Immunofluorescence was used to examine the expression of RSPO4 and fibrosis biomarkers. Cells were paraformaldehyde-fixed followed by permeabilization with 0.1% Triton X100 in PBS and blocked with 4% bovine serum albumin. Then cells were incubated with rabbit polyclonal antibody for RSPO4 (Abcam, USA), α-SMA (Abcam, USA), and collagen-I (Abcam, USA) overnight at 4 °C. Cells were then incubated with HRP-conjugated rabbit IgG (CST, USA) for 1 hour. Nuclei were stained with 4′,6-diamidino-2-phenylindole (Sigma-Aldrich, USA). Images were captured using an epifluorescence microscope (Olympus, Tokyo, Japan).

### PCR

The mRNA level was quantified by real-time PCR (StepOne-Plus, Applied Biosystems, CA) per the manufacturer’s instructions. To minimize the problems associated with DNA contamination, primers were designed to span at least one intron in the genomic sequence. Primers used for amplification are listed in Table 2. Total RNA from the cells was isolated using Trizol (Invitrogen, Carlsbad, CA) according to the manufacturer’s protocol. The quality of isolated RNA was assessed at absorbance ratios of A260/A280 (1.8–2.2) and A260/A230 (3.0–4.0) with a UV-1800 spectrophotometer (Shimadzu, Kyoto, Japan). Thermocycling conditions consisted of an initial step of 2 min at 50 °C, denaturation of 10 min at 95 °C, followed by 35–40 cycles at 95 °C for 30 s, 60 °C for 45 s, and 72 °C for 1 min and a final elongation step at 72 °C for 10 min. Each sample was analyzed in triplicate, with β-actin used for normalization. Comparative threshold (Ct) method was used for calculating the relative amount of mRNA of the sample compared with the control.

**Table 2:** Primer sequences.

Gene	Forward sequence	Reverse sequence
RSPO4	5‘-ATTCTGCTGGAGAGGAACGA-3‘	5‘-CTCCTGACACTTGGTGCAGA-3‘
α-SMA	5‘-CTGACAGAGGCACCACTGAA-3‘	5‘-CATCTCCAGAGTCCAGCACA-3‘
Collagen-I	5‘-TTCACCTACAGCACGCTTGTG-3‘	5‘-GATGACTGTCTTGCCCCAAGTT-3‘
β-actin	5‘-TCATCACTATTGGCAACGAGC-3‘	5‘-AACAGTCCGCCTAGAAGCAC-3‘

### Western blotting

Protein fractions were extracted using the NE-PER Extraction Reagent Kit (Pierce, United States) according to the manufacturer’s instruction. The extracts were then subjected to sodium dodecyl sulfate polyacrylamide gel electrophoresis for protein separation and then electrophoretically transferred to nitrocellulose membrane (Axygen, Union City, CA). After blocking by phosphate-buffered saline containing 5% fat-free milk, the nitrocellulose membranes were incubated with rabbit polyclonal antibody for RSPO4 (dilution, 1:1000; Abcam, USA), α-SMA (dilution, 1:300; Abcam, USA), collagen-I (dilution, 1:200; Abcam, USA), and β-Actin (dilution,1:5000; Abcam, USA) overnight at 4 °C and then incubated with HRP-conjugated rabbit IgG (CST, USA) for 1.5 h at room temperature. The immunolabeled proteins were detected using a commercial ECL detection kit (HarO, Shanghai, China).

### Lipid droplet analysis

Accumulations of lipid droplets in HSCs were determined using an Oil red O assay. In brief, 0.5 g of Oil red O powder (American Mastertech Scientific; CA, USA) was dissolved in 100 mL of isopropanol to make a stock solution, which was diluted with water (3:2) and filtered. Staining was performed at 25 °C for 1 h, and plates were then washed twice in ddH2O and observed and photographed under a microscope (CK Microscope, Olympus, Tokyo, Japan).

### HSC migration assay

A 24-well Transwell chamber with 8.0 µm pore size (Costar; Corning Incorporated, Corning, NY, USA) was used for the migration assay. The pre-balance of the Transwell chamber was performed by adding DMEM without FBS into the upper and bottom chamber overnight at 37°C. HSCs were trypsinized and suspended in 200 µl serum-free medium and were seeded in the upper chamber at a density of 2 × 10^5^ cells per well. The bottom chamber was filled with DMEM containing 10% FBS. Following incubation for 24 h at 37°C. Migrated cells were then dried, fixed with methanol, and stained with 0.1% crystal violet at room temperature for 10 min. The transmembrane cells were counted using a light microscope (OLYMPUS BX43, Olympus Co., Japan) under x100 magnification.

### HSC invasion assay

HSCs were seeded in a six-well Bio Coat Matrigel Invasion Chamber (Beckton-Dickinson, Bedford, MA, USA) at a density of 2 × 10^5^ cells per well. The bottom chamber was filled with DMEM containing 10% FBS. Following incubation for 24 h at 37°C, the non-invading cells were removed from the upper surface of the membrane by soft scratch with small cotton swabs. The membrane was then fixed with 4% Paraformaldehyde for 15 min and stained with 0.1% crystal violet at room temperature for 10 min. The invading cells were counted using a light microscope (OLYMPUS BX43, Olympus Co., Japan) under ×100 magnification.

### MTT cell proliferation assay

HSC proliferation was analyzed using the MTT (3-(4,5-dimethylthiazol-2-yl)-2,5-diphenyltetrazolium bromide) cell proliferation assay. HSCs were seeded into 96-well plate at the density of 2.0 × 10^3^ per well. Cell growth was measured by MTT assay at the 6 h, 12 h, 24 h, 48 h, and 72 h after cell seeding. In brief, 100 μg of MTT (Sigma, United States) was added into each well. After incubation for 4 h at 37 °C, purple formazan crystals generated from viable cells were dissolved by adding 100 μl DMSO (Sigma-Aldrich, USA) in each well. The absorbance of each well was then read at 570 nm wavelength in Bio-Rad 550 microplate reader (Bio-Rad, United States). HSCs growth rates were then calculated as in Eq. 1.

1 $$Cell\,growth\,rates = \left( {average\,absorbance\,of\,test\,group/control\,group} \right) \times 100\%$$

### Fecal sample collection

Rats were transferred to a second home cage for the collection of fecal pellets. Fecal pellets were collected, flash-frozen, and stored at −80 °C for later analysis.

### DNA extraction and 16 S rRNA gene amplicon sequencing

Fecal microbial DNA was extracted from 250 mg frozen sample using Power Fecal DNA Kit (QIAamp, Germantown, MD, USA) following the manufacturer’s instructions. The final DNA concentration and purification were determined by NanoDrop 2000 UV-vis spectrophotometer (Thermo Scientific, Wilmington, USA), and DNA quality was checked by 1% agarose gel electrophoresis. The extracted DNA was stored at –20°C for future processing.

The amplicon library was constructed by amplifying the V3/V4region of 16 S rRNA gene using the primer 338 F_806 R (338F: 5′-ACTCCTACGGGAGGCAGCAG-3′, 806R: 5′-GGACTACHVGGGTWTCTAAT-3′). PCR was performed by thermocycler PCR system (GeneAmp 9700, ABI, USA) with TransStartFastpfu DNA Polymerase AP221-02 by using the following conditions: 95 °C for 3 min followed by 30 cycles of 95 °C for 30 s, 60 °C for 30 s, and 72 °C for 45 s, and a final extension step at 72 °C for 8 min. The PCR products amplicons are quantified using QuantiFluor™ -ST (Promega, United States) according to the manufacturer’s protocol. Purified DNA samples were paired-end (PE300, 2∗300 bp) sequenced by using Illumina Miseq. Unqualified reads were removed to obtain clean data for further analysis. After clustering operational taxonomic units (OTU) at 97% identity, a total of 1971 OTUs were recovered from the fecal samples.

### 16 S rRNA sequence analysis

The sequenced 16 S reads were analyzed by using the QIIME software package^38^, the R programming language (R version 3.5.1), and the Python programming language (Python version 3.6). Data decontamination was performed using FLASH^39^ and Trimmomatic^40^. OTUs were created by clustering the reads at similarity threshold of 97% using Usearch (vsesion 7.0, http://drive5.com/uparse/). The taxonomy of each OTU representative sequence was analyzed by RDP Classifier (http://rdp.cme.msu.edu/) against the Bacteria and archaea 16 S rRNA gene databases Silva (Release128 http://www.arb-silva.de) using confidence threshold of 0.7. Indexes of α-diversity were calculated using mothur (version v.1.35.1 https://www.mothur.org/).

Circos plot was drawn by Circos-0.67-7 (http://circos.ca/), and a phylogenetic tree was inferred by using FastTree (version 2.1.3 http://www.microbesonline.org/fasttree/). Principal component analysis and PCoA analysis (Principal coordinates analysis) were performed by using R and python for analysis and plotting. With R Ade4 and cluster, enterotyping analysis calculated Jensen-Shannon Distance and PAM (Partitioning Around Medoids) according to the relative abundance of the species level first and then do clustering. Differences analysis was performed by using R for significant difference between groups (Kruskal–Wallis *H* tests) and Galaxy (http://huttenhower.sph.harvard.edu/galaxy/) for LEfSe.

### Statistical analysis

All statistical analyses were performed using R software (version 3.5.1). Data were presented as mean ± SEM. A value of *p* < 0.05 was considered to be statistically significant. A two-tailed Student’s *t* test was employed to evaluate the differences between groups. For semi-quantitative analysis of histological staging, nonparametric tests (Mann–Whitney *U* test or Kruskal–Wallis rank-sum test) were used.

### Reporting summary

Further information on research design is available in the Nature Research Reporting Summary linked to this article.

## Supplementary information

Supplementary Figures

Reporting Summary

## Data Availability

Source data that support the findings in our study are available upon reasonable request to the corresponding authors. In all, 16 S rRNA sequence data can be accessed from NCBI BioProject under identifier PRJNA543718. Original gel images corresponding to Fig. 1d are available in Supplementary Figure 2.

## Code availability

Our analysis used standard, open source software. R packages are available on CRAN (cran.r-project.org), Galaxy (usegalaxy.org), and the Python programming language (Python version 3.6).
