# Supplementary material for: RSPO4-CRISPR alleviates liver injury and restores gut microbiota in a rat model of liver fibrosis
Source: Commun Biol. 2021 Feb 18;4:230. doi: 10.1038/s42003-021-01747-5 (PMC7893072; doi:10.1038/s42003-021-01747-5)
Supplement: Supplementary file 1 — Supplementary Figures [file 42003_2021_1747_MOESM1_ESM.pdf]

Supplementary figures

Supplementary Figure 1

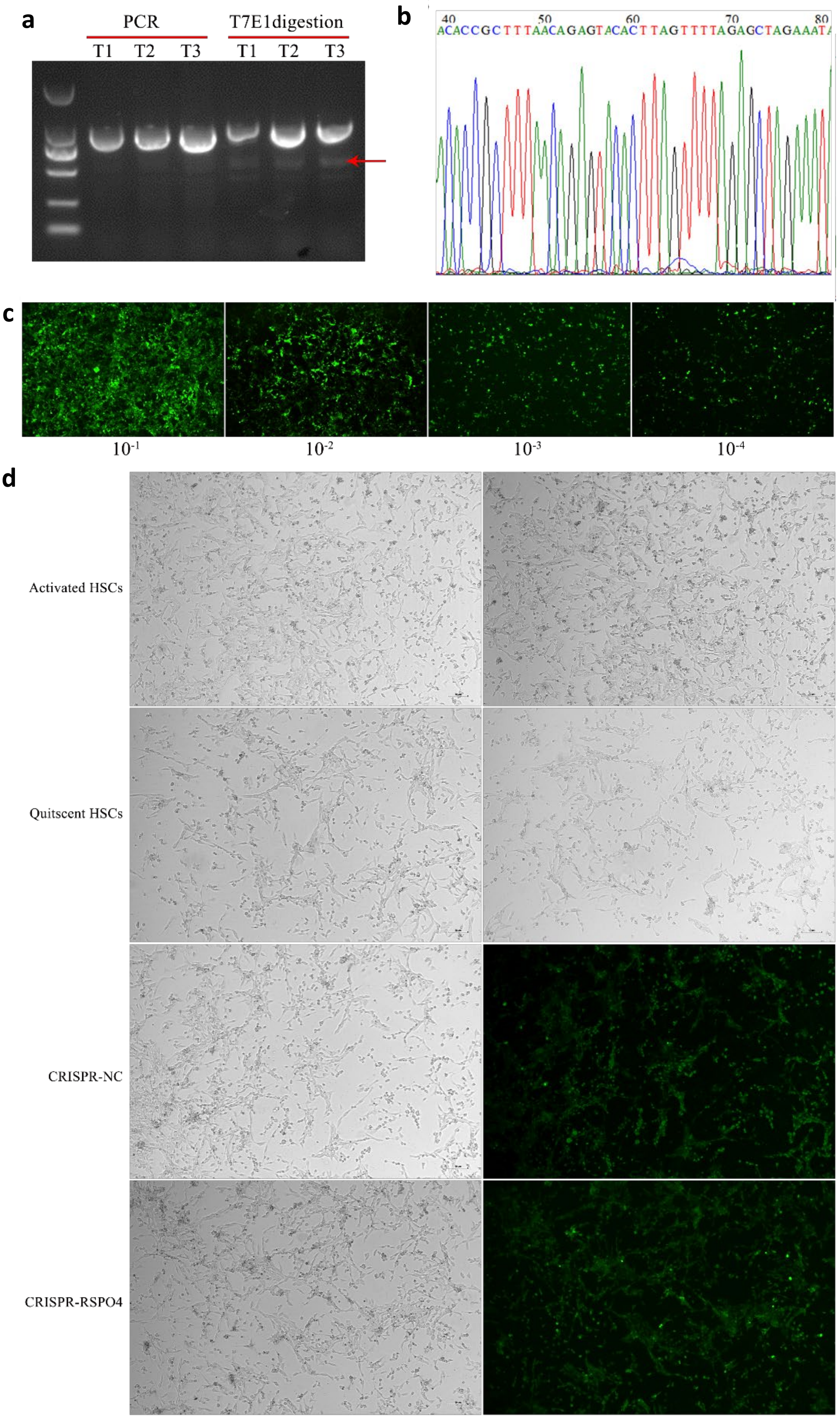

Supplementary Figure 1. Lentiviral vector expressing RSPO4-CRISPR was successfully constructed. a. The result of T7EI enzyme digestion indicated the mutation of an RSPO4 target site. **b.** Results of sequencing indicated CRISPR-T1 vector including the target sequence GCCAGTGCCAAGGCAACCGA. **c.** Lentiviral vector titers were determined by fluorescence-activated cell sorting analysis. **d.** HSCs transfected with CRISPR-RSPO4.

Supplementary Figure 2

a. RSPO4

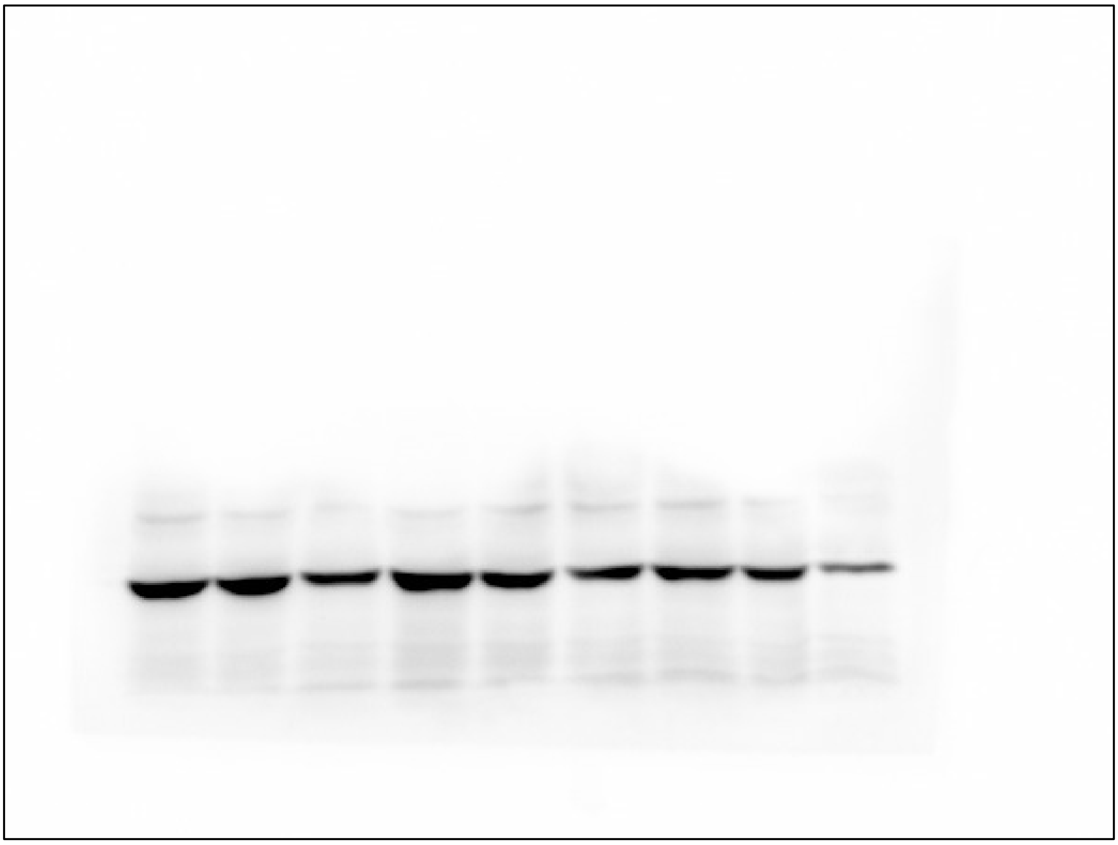

b. α-SMA

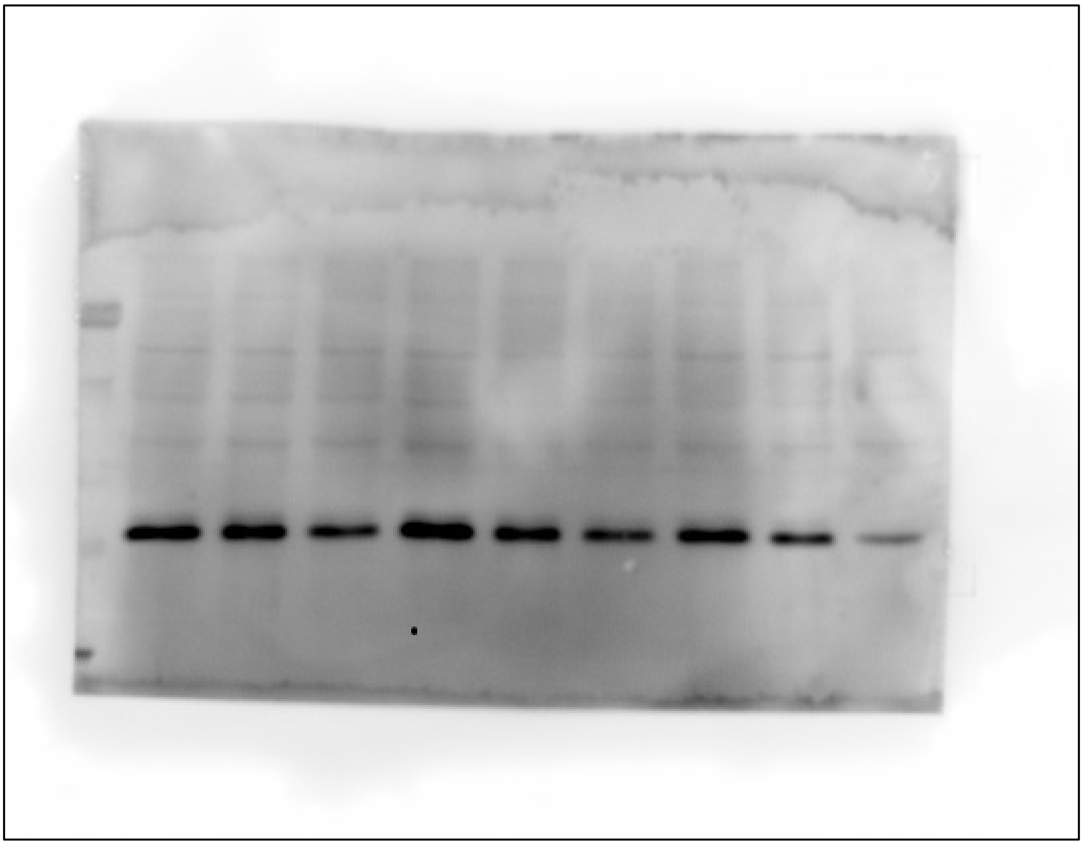

c. Collagen-I

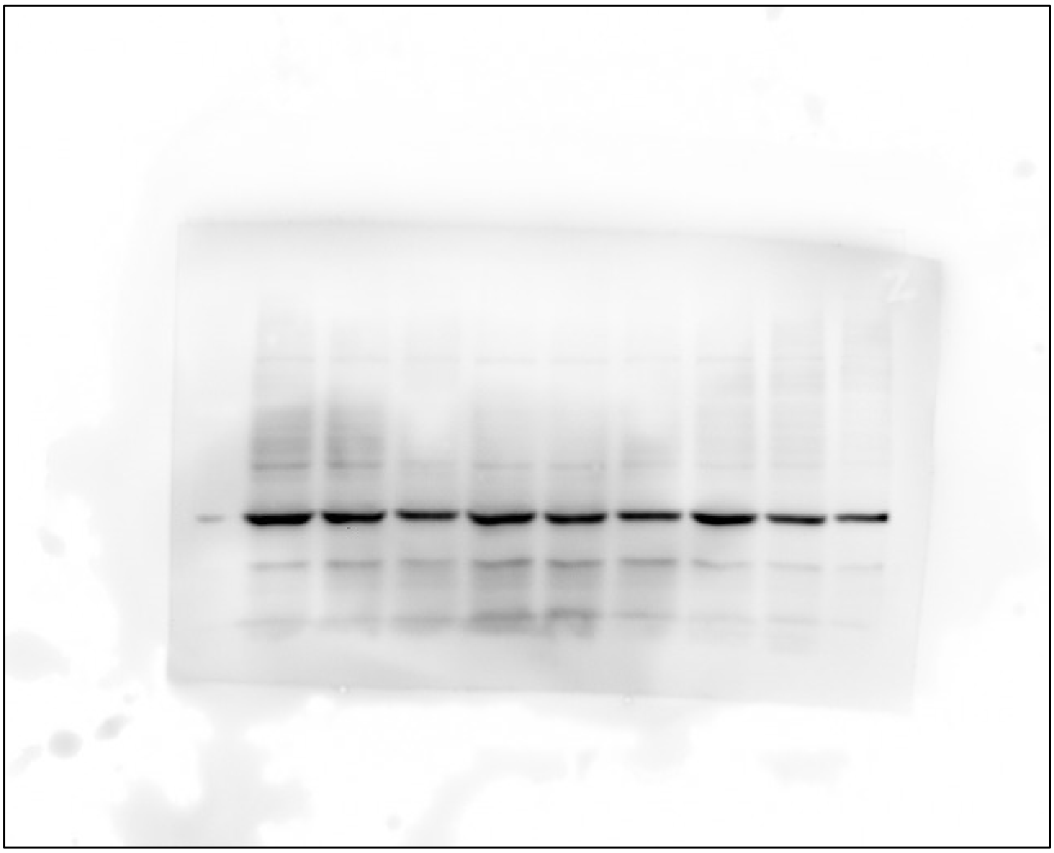

d. β-actin

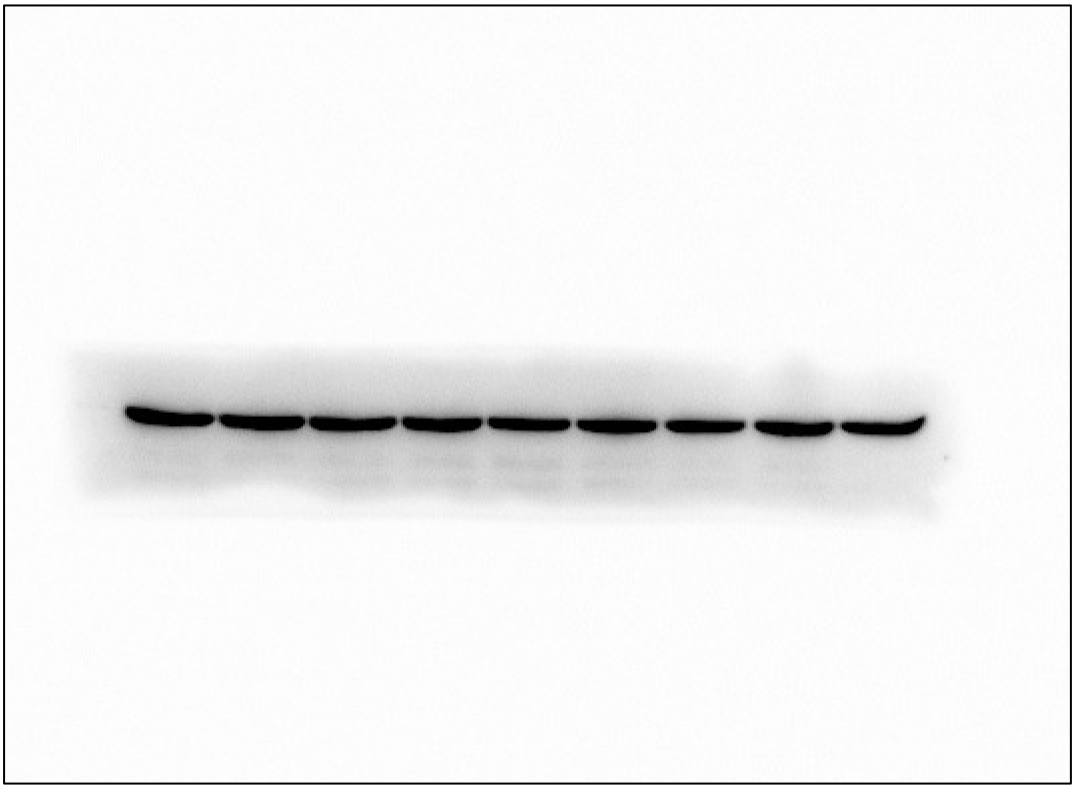

Supplementary Figure 2. Original gel images from Figure 1d.
